# The Pertinent Role of Cell and Matrix Mechanics in Cell Adhesion and Migration

**DOI:** 10.3389/fcell.2021.720494

**Published:** 2021-10-13

**Authors:** Claudia Tanja Mierke

**Affiliations:** Faculty of Physics and Earth Science, Peter Debye Institute of Soft Matter Physics, Biological Physics Division, University of Leipzig, Leipzig, Germany

**Keywords:** cell and matrix mechanics, constraints, extracellular matrix, alignment, viscoelasticity, stiffness, endothelium, immune cells

## Introduction

The processes of cell adhesion and migration fulfill crucial tasks during physiological developmental processes, immune responses, angiogenesis, or tissue injury and pathological diseased states including the cancer, cancer angiogenesis and its malignant progression, referred to as metastasis. Thereby, the cells undergo tremendous alterations that obviously lead to pronounced morphological alterations that are closely coupled to intracellular structural changes and mechanical perturbations (Humphrey et al., 2002; Guck et al., 2005; Mierke et al., 2008a, 2011; Kumar and Weaver, 2009; Menon and Beningo, 2011; Lekka et al., 2012; Humphries et al., 2019; Mierke, 2019a). These alterations occur on different cellular length scales, such as bulk alterations, compartmental alterations, structural compositional changes, molecular alterations down to gene expression regulatory events. All of these types of changes cannot be treated as purely separate events that can be fully deciphered in an independent manner. Hence, the specific microenvironmental constraints play a prominent role in unraveling the impact of the intricate interplay. Due to the vastly high number of molecules that function in cell adhesion under physiological and pathological processes, the agglomeration of proteins within focal adhesion has been termed cancer cellular adhesome to discriminate them from randomly distributed surrounding proteins (Maziveyi and Alahari, 2017). The contributing proteins of the adhesome can be divided into four different branches of the basic adhesion system which includes the Talin-Vinculin (Mierke et al., 2008a, 2010; Golji et al., 2011; Wang et al., 2019; Boujemaa-Paterski et al., 2020), FAK-Paxillin (Hu et al., 2015; Mierke et al., 2017; Ripamonti et al., 2021), α-Actinin-Zyxin-VASP (Oldenburg et al., 2015), and ILK-PINCH-Kindlin biochemical signal transduction pathways (Honda et al., 2013; Horton et al., 2015; Kunschmann et al., 2017). All of them represent critical pathways or mechanosensory systems to respond to changes in the mechanical homoestatic stage of cells. There are also additional organizational structures such as protrusions, podosomes, invadosomes, and similar structures for the motile function or migratory capacity of cells. When these structures, such as invadopodia are altered, the process of cancer metastasis can be impaired, such as for melanoma cells (Karamanou et al., 2021). Cancer cells act in various types of directional cell migration compromising chemotaxis (chemoattractant gradient), haptotaxis (environmental gradient), electrotaxis (ionic flux), galvanotaxis (electrical attractant), pilotaxis, and durotaxis (rigidity attractant) (Roussos et al., 2011; Allen et al., 2013; Mierke, 2021). Recognition of the microenvironment by cancer cells allows them to translate diverse signaling conveyed through focal adhesions. The stiffness produced by the extracellular matrix initiates and synergizes with the cell-matrix forces imposed by the cells (Krieg et al., 2008). Cells are able to capture multiple properties of the extracellular matrix in terms of stiffness and analysis of anisotropy (Geiger et al., 2009).

## New Frontiers in the Field of Cell Adhesion and Migration

In the field of cell adhesion and migration, the impact of the extracellular matrix structure on the motility of single or grouped cells has been investigated quite well-during the last decade. However, the mechanical cues of the microenvironment on the adhesive, migratory, and functional tasks of cells or tissues has not yet been addressed on a broad scale (Mierke, 2020). Instead, it has been largely explored how alterations in cellular metabolism influence cell adhesion and migration. Recently, it has been stated that cellular metabolism and matrix mechanics are coupled (Levental et al., 2009; Tilghman et al., 2012; Chaudhuri et al., 2014; Ge et al., 2021). Moreover, it needs to be revealed whether there exist even similarities between the migration and invasion of individual or grouped cells in terms of the impact of mechanical characteristics. It has turned out that the cell mechanical characteristics can either foster or impair the adhesion and migration processes, what can be considered as one of the biggest grand challenges in this field (Bergert et al., 2012; Mierke, 2013; De Pascalis and Etienne-Manneville, 2017). The same holds true for the matrix mechanics or confinement on cell migration and invasion (Mierke et al., 2011; Mierke, 2014; Winkler et al., 2020; Kiran et al., 2021). In specific detail, the viscoelasticity has emerged as a key characteristic feature of cellular behavior, overall cellular shape, morphology and function of whole tissues (Clément et al., 2017; Tan et al., 2018; Barriga and Mayor, 2019; Chaudhuri et al., 2020). Thus, the field of cell adhesion and migration needs to cover also cell mechanical aspects as well as matrix mechanical aspects.

Besides the migration and invasion of individual cells, the collective cell migration plays a prominent role in a number of physiological processes, including wound healing and embryogenesis, and in several pathological processes, such as cancer metastasis. Although there is abundant experimental and theoretical evidence (Haeger et al., 2014; Pajic-Lijakovic and Milivojevic, 2019; Lohmann et al., 2020; Mitchel et al., 2020), the unifying mechanism governing collective cell migration is not best identified. However, there may also remain differences in the collective motion of cells, such as the tricellular junctions, which appear and mature at vertexes where three cells come together. Moreover, they are an ideal place to guide and govern the shape of cells and coordination of the multicellular migration (Lohmann et al., 2020). However, their function in epithelial tissue dynamic remains poorly defined. Most investigations have examined engineered model wounds to analyze collective cell migration in an epithelial solitary layer. These synthetic model wounds have a high-density of cells relative to physiological scenes such as a wound healing setting, where cell damage occurred from applied incision, and cancer metastasis settings, where smaller cell clusters are commonly involved. Based on these findings, the two systems may not be fully related, and further studies are necessary to better comprehend collective cell migration patterns in physiological scenes, which turns out to be a new frontier in cell migratory research. In specific, even the expansion of tissues can be restricted by strong confinements (Kiran et al., 2021). In addition, the migration of a collection of cells needs to be subdivided in areas of high, intermediate or low migration can be related to mechanical cues. Despite confinement, collective cell migration is more efficacious than individual cell migration by virtue of its intercellular forces (De Pascalis and Etienne-Manneville, 2017).

**Figure 1:**
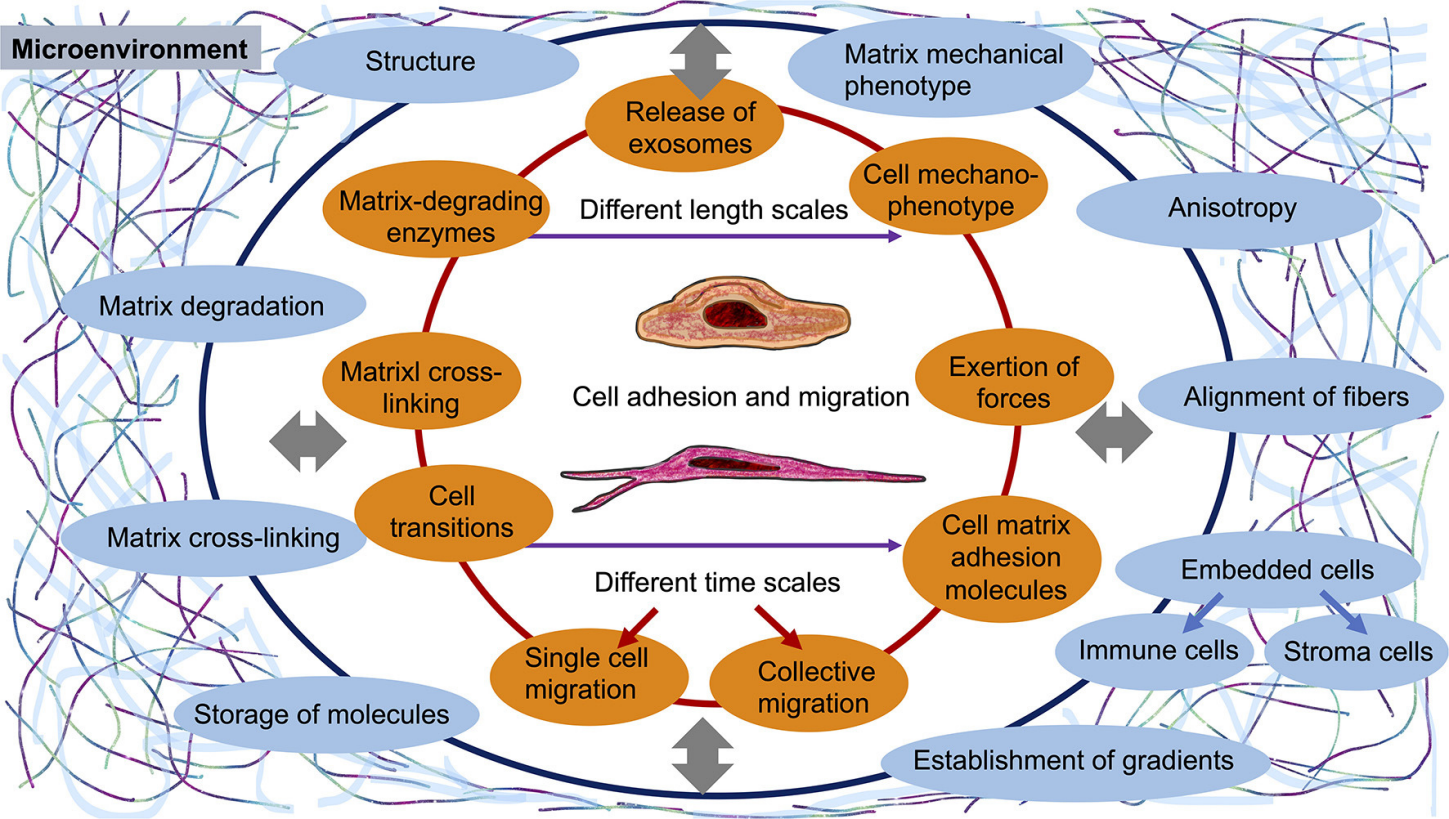
Existing interactions between cells or a collection of cells and the microenvironment during the adhesion and migration of cells. The current gaps represent new frontiers for cell adhesion and migration research. There is a mutual interplay between cells and their surroundings that takes place at vastly different length scales and depends dynamically on time (different time scales).

Apart from the pure cell mechanics, the environment is a prominent regulator of cell and tissue mechanics that needs to be regarded as well (Pokki et al., 2021). Thereby the interaction between cells can take place on different levels, such as the macroscopic level, including the bulk cell and tissue mechanical level, the mesoscopic level, encompassing structural alterations of extracellular matrices through degradation (Kessenbrock et al., 2010; Slattery et al., 2013; Grolman et al., 2020; Winkler et al., 2020; Curtis et al., 2021) or cross-linking processes (Levental et al., 2009) and the storage of growth factors, cytokines, chemokines, cell adhesion receptor ligands or enzyme-inhibiting molecules and the microscopic level, including fiber mechanics and cross-linker protein mechanics. On the microscopic scale, the organelles of cells, such as the nucleus, nucleolus, mitochondria, endoplasmic reticulum, Golgi apparatus and lysosomes have an additional effect on cell adhesion and migration, since these compartments may represent major steric obstacles for cell migration (Friedl et al., 2011; Janel et al., 2019; Krause et al., 2019; Efremov et al., 2020; Fischer et al., 2020; Zuela-Sopilniak et al., 2020), which can be broken down by rupture or elevated by fusion among organelles, such as mitochondria. The connection between cell, matrix and tissue mechanics on different length scales and dynamically on different time scales seems to be an additional new frontier in cell adhesion and cell migratory research (Figure 1).

Besides cell mechanical cues, the release of exosomes, microvesicles and apoptotic bodies from cancer cells can impact their migratory and invasive capacity and phenotype, as they have been shown to regulate cell adhesion and migration (Antonyak et al., 2011; Yoon et al., 2014; Sedgwick et al., 2015; Ståhl et al., 2019). Consequently, their effect on matrix mechanics, and in turn on their own regulation by matrix mechanical cues, appears to be evolving and forming a potential new frontier for cell adhesion and migration. Delivery of specific substances to recipient cells, such as matrix embedded cells or neighboring cancer cell subpopulations, generally occurs through the uptake of exosomes or microvesicles and may alter the mechanical and structural phenotype of the target cell. Finally, the intercellular communication has been altered as a result.

Apart from the mechanics of these extracellular elements, matrix embedded living cells, such as stroma fibroblasts or vessel-lining endothelial cells with or without pericytes can modify the whole scene of cell adhesion and migration on all length scales in terms of mechanical cues. In specific, these cells can alter their own mechanical characteristics and functions to either indirectly affect the adhesion and migration properties of targeted cells through changes of the local microenvironment or directedly release substances that then stimulate a structural or mechanical response of these cells. The connection to other embedded cells and their impact on cell mechanical properties represents another new frontier in this field.

It can be asserted that the interaction of various cell types or the interference of cells with their close microenvironment depends on the mechanical phenotype of each associated element. Thus, the focus demands to be shifted toward the mechanics of cells, nearby cells and extracellular matrix. The investigation of the mechanical cues will encourage the sharpening of the focus spot and provide a clear character.

## Inclusion of Immune Cells as Potentially Occasional Adherent Cells

Apart from stroma cells, such as tissue fibroblasts, immune cells can affect the mechanical properties of the extracellular matrix microenvironment (Hynes and Naba, 2012; Cox et al., 2013; Gonzalez et al., 2018; Pakshir et al., 2019; D'Urso and Kurniawan, 2020) and other nearby cells, such as cancer cells (Fiegl et al., 2006; Zhan et al., 2017; Zheng and Li, 2020). Thus, these immune cell types, encompassing γδT-cells, B-lymphocytes, tumor-associated macrophages, CD8+ and CD4+ lymphocytes, natural killer cells, dendritic cells, T-helper-1 (Th1) cells, Th9 cells and M1 macrophages, T-regulatory (Treg) cells, N1 and N2 neutrophils and cancer associated eosinophiles need to be included in this scenario of cell adhesion and migration (Stankovic et al., 2019; Grisaru-Tal et al., 2020). Thereby it requires the discrimination between the various types of immune cells and their individual impact on matrix and neighboring cell mechanics. The interplay of these different subsets of immune cells are crucial in challenging the overall mechanical phenotypes of cells and tissues. There may also be raised the question whether specific mechanical cues can induce an adherent state of distinct immune cells on their way through tissue microenvironment.

## New Goals for Cell Adhesion and Migration

Since the impact of embedded cells on 3D migration and invasion, the role of the mechanical characteristics of cells and their microenvironment including its bi-directional interplay have been recognized, they all can be determined as new frontiers that have been identified on individual and collective migration. Consequently, the goals for future cell adhesion and migration efforts are the knowledge of the role of embedded cells, such as stroma cells and immune cells, the effect of mechanics on cell adhesion and motility and its mutual interplay including the release of exosomes and microvesicles. However, it is not yet entirely obvious how viscoelasticity alters the functional phenotype of cells, but it is definitely an encouraging new frontier. In particular, the biophysical technologies are yet to be improved and advanced. Hence, this specialty grand challenge article focusses in the following section on some crucial technologies employed to analyze the viscoelasticity of cells and extracellular matrices. It also illustrates how the cellular mechanophenotype is essential to identify, characterize, and therapeutically treat a variety of diseases such as cancer, tissue injury, acute or chronic inflammation, or fibrotic diseases. Finally, a future perspective on the importance of viscoelasticity of cells and matrices is presented.

## Development, Establishment, and Advancement of Biophysical Techniques

Firstly, there is still an urgent need for the development of novel biophysical techniques and analytical procedures to measure the mechanical properties of individual cells, collection of cells and tissues. Thereby, statistical analyses need to be involved due to the broad heterogeneity of cell populations. Secondly, the further development of existing biophysical techniques, such as super-resolution microscopy or microrheological techniques or microfluidic approaches seems to be highly promising in addressing the mechanical feature of cell migration. Thirdly, the assessment of the microenvironmental physical deformation of cells or collections of cells seems to be key for revealing the interplay between specific cell types and their extracellular matrix environment. Thereby, the dynamically evolving matrix displacement, referred to as flow displacements are highly required.

Super-resolution microscopy, a technique that relies on single-molecule spatial information localization, comprises photoactivation localization microscopy (PALM), stochastic optical reconstruction microscopy (STORM), ground state depletion microscopy followed by individual molecular return (GSDIM) and universal point accumulation imaging in the nanoscale topography (uPAINT) (Godin et al., 2014; Paszek et al., 2014). iPALM represents a truly singular type of super-resolution microscopy that couples PALM with concurrent multiphase photon interferometry of single fluorescent molecules (Betzig et al., 2006). This type of imaging technology can be applied to visualize fluorescently labeled focal adhesion proteins with three-dimensional nanoscale spatial resolution (Kanchanawong et al., 2010). The improvement of these techniques can help to address the intracellular organelle level including the fission and fusion of organelles that can alter cell mechanical properties and subsequently cell adhesion and migration behavior.

Microrheological approaches, such as Magnetic tweezers (Kollmannsberger and Fabry, 2007; Sarkar and Rybenkov, 2016), Magnetic twisting cytometry, and Single article tracking approaches are both still state-of-the-art biophysical techniques that can be refined by including temperature issues (Aermes et al., 2021), cytoskeletal coupling or membrane receptor strengths to characterize the mechanophenotype of cells.

When addressing the mechanical properties of cells without the impact of cell adhesion, improved cell deformation/stretching biophysical techniques can be used, compromising an optical cell stretcher (Guck et al., 2005; Mierke et al., 2017, 2020; Mierke, 2019b) and microfluidics-based cell stretcher that represents a channel confinement for directional flowing or migrating cells (Huang et al., 2020; Yao et al., 2020).

Other even more famous biophysical techniques are atomic force microscopy (AFM) (Fischer et al., 2020), Micropipettes or Dual Micropipettes (González-Bermúdez et al., 2019), which the latter to be employed for intercellular adhesion force analyses. Last, but not least, the analysis of matrix displacement by cellular forces, such as Flow displacement technique in 3D, and traction force techniques in 2D, 2.5 D and 3D require to be further developed and combined with biological techniques (Mierke et al., 2008b; Legant et al., 2013; Cóndor et al., 2017; Hazlett et al., 2020). These dimensions can be expanded to 4D by adding time as fourth dimension. These forces may help to enlighten the impact of internal mechanotransduction events on cell adhesion and migration and vice versa the external mechanotransduction through other neighboring cells.

## Future Directions and Conclusions

There is no other way in the future then that of combined cell biological and biophysical research in the broad field of cell adhesion and migration. The coupling of cell adhesion and migration to specific diseased states and non-equilibrium states seems to be required and promising. The transition of cellular states, such as individual cells from epithelial to mesenchymal or intermediate states, or such of a collection of cells from jamming to unjamming including intermediate states, are crucial feature for understanding under which structural or mechanical constraints cells or collections of cells start to move. Thereby the formation of adhesomes compromising all four branches plays a critical role and requires the connection of cellular and developmental biophysics to systems biological or genetic approaches. All the gained knowledge on the impact of the 3D cellular microenvironment and in specific the extracellular matrix scaffold can be employed to develop beyond state-of-the art theranostic approaches and propose new smart biomaterials.

## Author Contributions

CM contributed conception, design, wrote the manuscript, and prepared the figure herself.

## Conflict of Interest

The author declares that the research was conducted in the absence of any commercial or financial relationships that could be construed as a potential conflict of interest.

## Publisher's Note

All claims expressed in this article are solely those of the authors and do not necessarily represent those of their affiliated organizations, or those of the publisher, the editors and the reviewers. Any product that may be evaluated in this article, or claim that may be made by its manufacturer, is not guaranteed or endorsed by the publisher.
